# Highly Dispersed Nickel-Containing Mesoporous Silica with Superior Stability in Carbon Dioxide Reforming of Methane: The Effect of Anchoring

**DOI:** 10.3390/ma7032340

**Published:** 2014-03-19

**Authors:** Wenjia Cai, Lin Ye, Li Zhang, Yuanhang Ren, Bin Yue, Xueying Chen, Heyong He

**Affiliations:** Department of Chemistry and Shanghai Key Laboratory of Molecular Catalysis and Innovative Materials, Collaborative Innovation Center of Chemistry for Energy Materials, Fudan University, Shanghai 200433, China; E-Mails: 11110220037@fudan.edu.cn (W.C.); 09110220051@fudan.edu.cn (L.Y.); lily_zhang@fudan.edu.cn (L.Z.); 09110220018@fudan.edu.cn (Y.R.); xueyingchen@fudan.edu.cn (X.C.)

**Keywords:** methane, dry reforming, Ni, mesoporous silica, anchoring effect

## Abstract

A series of nickel-containing mesoporous silica samples (Ni-SiO_2_) with different nickel content (3.1%–13.2%) were synthesized by the evaporation-induced self-assembly method. Their catalytic activity was tested in carbon dioxide reforming of methane. The characterization results revealed that the catalysts, e.g., 6.7%Ni-SiO_2_, with highly dispersed small nickel particles, exhibited excellent catalytic activity and long-term stability. The metallic nickel particle size was significantly affected by the metal anchoring effect between metallic nickel particles and unreduced nickel ions in the silica matrix. A strong anchoring effect was suggested to account for the remaining of small Ni particle size and the improved catalytic performance.

## 1. Introduction

Carbon dioxide reforming of methane (DRM) has received considerable attention, as it is a promising way to utilize natural gas and to reduce greenhouse gases [1–4]. The H_2_ and CO formed in this reaction has a low H_2_/CO ratio, which is suitable in the synthesis of valuable long-chain hydrocarbons and oxygenated chemicals through the Fischer–Tropsch process [5–7]. Furthermore, this reaction can also be applied in a chemical energy transmission system based on its strong endothermic characteristic [8]. However, the dry reforming is prevented from commercialization, due to the absence of economical and effective catalysts operated efficiently under severe reaction conditions with good stability.

Although noble metal-supported catalysts (Rh, Ru, Pd, Pt, Ir) are found to have promising catalytic performance in terms of conversion and selectivity for DRM, the high cost of noble metals makes them a less than ideal choice [9,10]. Nickel is considered a good replacement for noble metals, due to its comparable catalytic performance and low cost. However, nickel-based catalysts are more easily deactivated, because of carbon deposition and active metal species sintering [11–13]. Numerous efforts have been devoted toward improving the catalytic properties of nickel-based catalysts. Ni/Al_2_O_3_ catalysts promoted with MgO and CeO_2_ appeared to be more resistant to carbon deposition in DRM due to the high Ni dispersion and low dehydrogenation activity [14]. The improved catalytic behavior of Ni/Ce-SBA-15 could be attributed to the incorporation of cerium into the framework of SBA-15, which promoted the dispersion of nano-sized Ni species and inhibited the carbon formation [15]. Wang *et al*. [16] reported that the mesoporous Ni-Al_2_O_3_ catalyst prepared by the one-pot method presented better long-term stability than that of the Ni-impregnated one. The enhancement of the catalytic stability was closely associated with the stabilization of the active nickel particles by alumina support. Liu *et al*. [17] found that the high conversion and catalytic stability over Pt-impregnated Ni-MCM-41 catalyst were due to the strong interaction between Pt and nickel species within the SiO_2_ matrix.

The previous experimental and theoretical studies confirmed that smaller Ni particles have a better ability to suppress the carbon deposition [2,18,19]. Nevertheless, confining the Ni particle size within the nanoscale dimension is difficult, because the sintering of the Ni particles easily takes place under the severe reaction conditions of DRM [20,21]. Recently, it was reported that the anchoring effect, a concept that was described by Yermakov [22], can facilitate the formation of the active Ni nano-clusters with high dispersion under the reaction condition [1,23,24]. Partially reduced Mo-Pt and Mo-Pd catalysts possessed better catalytic activity in ethane hydrogenolysis in comparison with the single metallic system, due to the higher dispersion and stability of metallic Pt and Pd anchored to the partially reduced Mo [22]. Quek *et al*. [25] reported that the anchoring effect between grafted nickel species and the TUD-1 support restricted the migration of nickel clusters, hence preventing the formation of large nickel particles. For the nickel-based bimetallic catalysts, Ni-Zr-MCM-41, the strong anchoring effect and the partial activation of CO_2_ by Zr^4+^ accounted for the high activity and long-term stability of the catalysts [23].

In the present work, mesoporous Ni-SiO_2_ samples were co-synthesized via the evaporation-induced self-assembly method. The catalytic behaviors for carbon dioxide reforming of methane over reduced Ni-SiO_2_ catalysts were investigated. The anchoring effect between metallic Ni particles and unreduced Ni ions within a silica matrix limits the growth of Ni particles and increases its dispersion, improving the stability and activity of the catalysts.

## 2. Results and Discussion

### 2.1. Physicochemical Properties of the Catalysts

**Figure 1.**
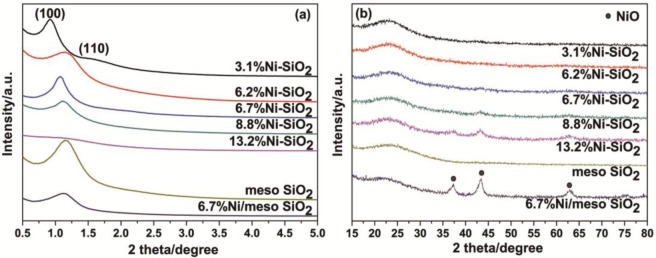
Small-angle (**a**) and wide-angle (**b**) XRD patterns of the meso-SiO2, Ni/meso-SiO2 and Ni-SiO2 samples.

**Figure 2.**
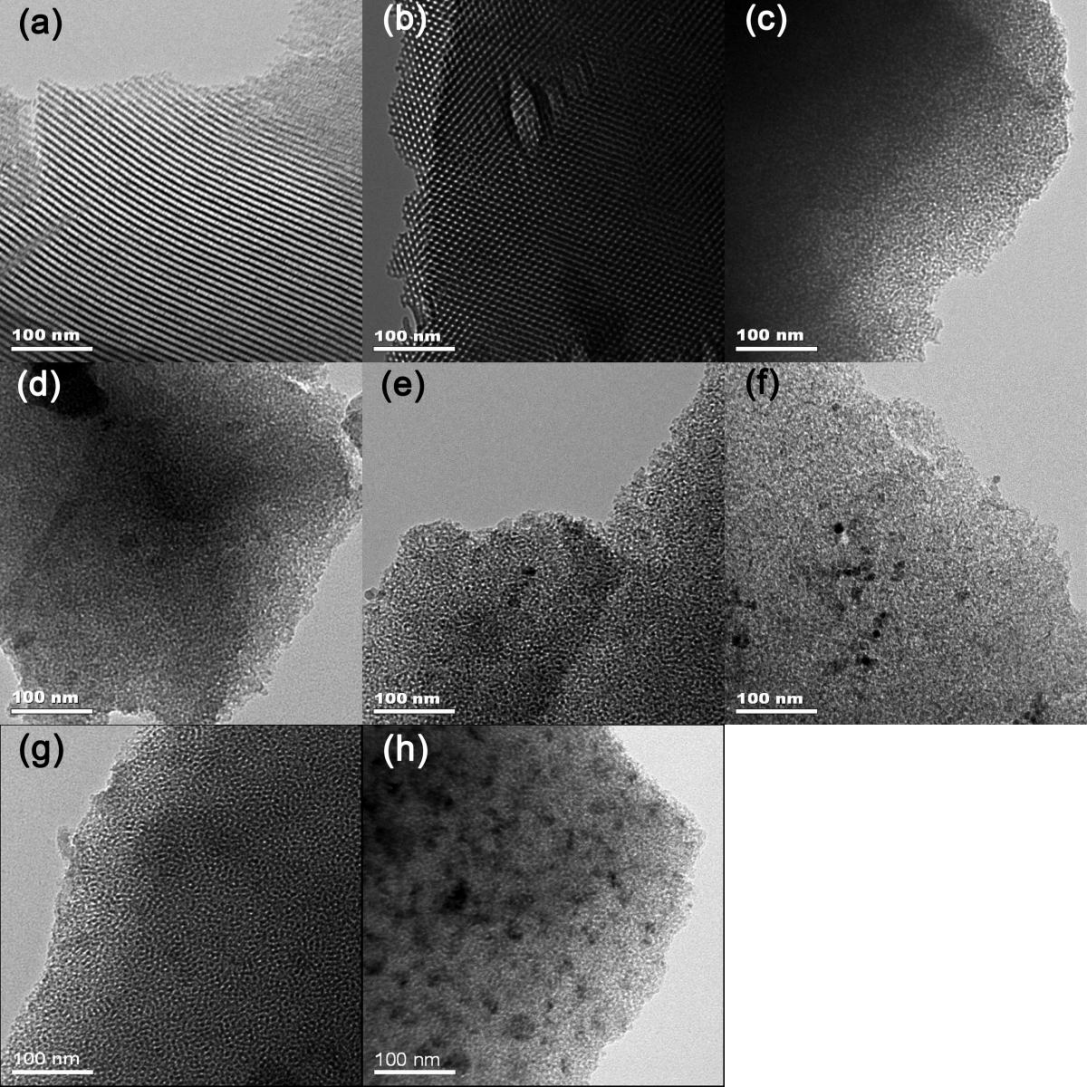
TEM images of (**a**,**b**) 3.1%Ni-SiO_2_; (**c**) 6.2%Ni-SiO_2_; (**d**) 6.7%Ni-SiO_2_; (**e**) 8.8%Ni-SiO_2_; (**f**) 13.2%Ni-SiO_2_; (**g**) meso-SiO_2_; and (**h**) 6.7%Ni/meso-SiO_2_.

Figure 1 depicts the XRD patterns of Ni-SiO_2_ samples with different Ni contents along with the Ni/meso-SiO_2_ prepared with the normal impregnation method. 3.1%Ni-SiO_2_ shows a strong (100) peak, along with a weak (110) peak, indicating a two-dimensional hexagonal structure, which agrees well with the results observed from TEM images in Figure 2a,b. The (110) diffraction peak at 2θ of 1.6° disappears as the nickel content increases, indicating that the ordering of the mesoporous structures of the Ni-SiO_2_ sample is declined. For 6.2%Ni-SiO_2_, 6.7%Ni-SiO_2_ and 8.8%Ni-SiO_2_, only one intense (100) peak in the region of 1.0°–1.2° is observed, and a wormlike mesoporous structure can be deduced (Figure 2c–e). 13.2%Ni-SiO_2_ does not show any peaks in the small angle region; this indicates that high nickel content leads to the disordered mesoporous structure (Figure 2f). As shown in Figure 1 and Figure 2g, meso-SiO_2_ shows a wormlike mesoporous structure. In addition, the wide-angle XRD patterns of all samples exhibit a broad peak around 23°, which suggests an amorphous structure of the framework. No obvious diffraction peak of nickel oxide can be identified for 3.1%Ni-SiO_2_ and 6.2%Ni-SiO_2_, implying that most of the nickel species are in the silica framework or highly dispersed on the silica surface. This is in agreement with the observation over Ni-MCM-41 reported in the literature [23]. Three diffraction peaks of (111), (200) and (220) belonged to NiO (JCPDS 78-0643) appear in 6.7%Ni/meso-SiO_2_ and 13.2%Ni-SiO_2_, indicating the formation of isolated NiO crystals. For 6.7%Ni-SiO_2_ and 8.8%Ni-SiO_2_, weak diffraction peaks of (200) can be observed.

**Figure 3.**
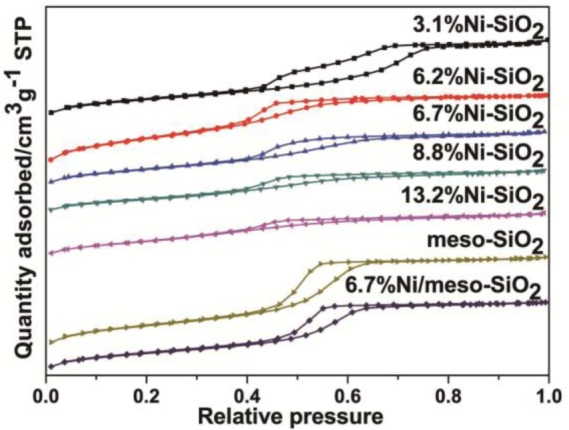
N_2_ adsorption-desorption isotherms of the meso-SiO_2_, Ni/meso-SiO_2_ and Ni-SiO_2_ samples.

The N_2_ adsorption-desorption isotherms of the meso-SiO_2_, Ni/meso-SiO_2_ and Ni-SiO_2_ samples are shown in Figure 3. All isotherms exhibit typical IV-type isotherms with a sharp increase of nitrogen uptake in the relative pressure range of 0.4–0.7, which is caused by capillary condensation of nitrogen inside uniform mesopores [1]. The textural properties of the samples are listed in Table 1. The decreased surface area and pore volume coincide with the increase of nickel loading. However, the surface area of 13.2%Ni-SiO_2_ deviates from the change trend, which is caused by the disordered mesoporous structure under high nickel content.

**Figure 4.**
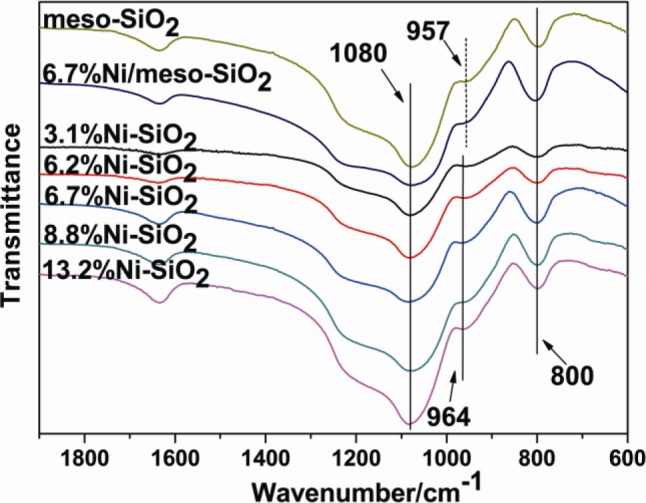
FT-IR spectra of the meso-SiO_2_, Ni/meso-SiO_2_ and Ni-SiO_2_ samples.

The Fourier-transformed infrared (FT-IR) spectra of the meso-SiO_2_, Ni/meso-SiO_2_ and Ni-SiO_2_ samples (Figure 4) show the symmetric stretching vibration band at around 800 cm^−1^ and the anti-symmetric vibration band at around 1080 cm^−1^ for the tetrahedral SiO_4_ structural units [26,27]. In addition, a band at 957 cm^−1^ can be observed in the meso-SiO_2_ and 6.7%Ni/meso-SiO_2_ samples. This band shifts to 964 cm^−1^ in Ni-SiO_2_ samples. Furthermore, the band intensity slightly increases with the increase of Ni content. The band at around 960 cm^−1^ has been widely used to characterize the incorporation of metal ions in the silica framework as the stretching Si-O vibration mode perturbed by the neighboring metal ions [28,29]. According to Liu *et al*. [30], this band is believed to be the proof of Ni atoms incorporating into the pore walls of Ni-KIT-6 samples. Furthermore, the change in intensity of this band was explained by the formation of nickel phyllosilicate on Ni@SiO_2_ catalyst [31]. This evidence indicates the presence of the isomorphous substitution of Si by Ni ions in Ni-SiO_2_ samples.

**Figure 5.**
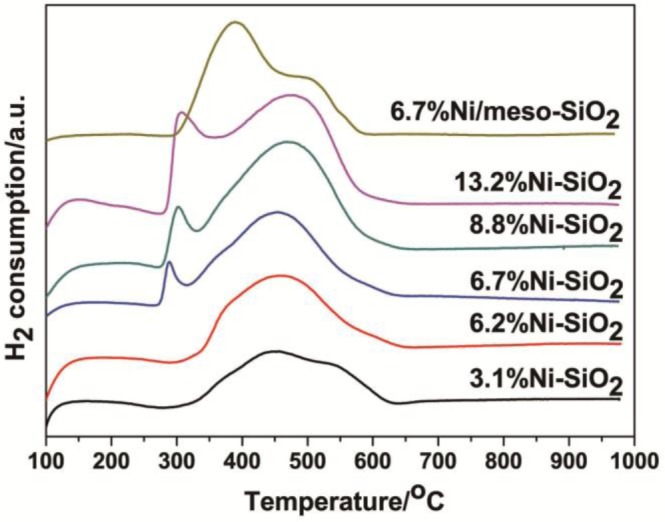
H_2_-TPR profiles of the Ni/meso-SiO_2_ and Ni-SiO_2_ samples.

Figure 5 shows the temperature-programmed reduction (TPR) profiles of the Ni/meso-SiO_2_ and Ni-SiO_2_ samples. For all Ni-SiO_2_ samples, one broad reduction peak centered at around 450–470 °C is observed. This can be explained by the reduction of nickel ions located from the surface to interior silica matrix, as well as the highly dispersed nickel oxide on the surface [1]. With the increase of nickel content, a low-temperature peak is observed in 6.7%Ni-SiO_2_, 8.8%Ni-SiO_2_ and 13.2%Ni-SiO_2_, which may be assigned to the reduction of bulky isolated nickel oxide particles [1,30]. This peak is shifted from 287 °C in 6.7%Ni-SiO_2_ to 308 °C in 13.2%Ni-SiO_2_, indicating that larger and worse dispersed isolated nickel oxide particles are formed outside the silica matrix in the latter sample [25,30]. To gain a better understanding of the reducing ability of the samples, the fraction of nickel species corresponding to the low-temperature peaks around 300 °C for 6.7%Ni-SiO_2_, 8.8%Ni-SiO_2_ and 13.2%Ni-SiO_2_ was estimated as shown in Table 1. The H_2_ consumed on 6.7%Ni-SiO_2_ in the low temperature region is 7.1% of the total amount of H_2_ consumed, which is increased to 23.8% on 13.2%Ni-SiO_2_. An increase of the H_2_ consumption in the low temperature region accounts for increasing the amount of isolated nickel oxide particles, which may convert to metal Ni particles. For 6.7%Ni/meso-SiO_2_, a strong peak at 388 °C and a shoulder at 494 °C are observed. Compared to Ni-SiO_2_ samples, the results indicate that majority of Ni species in 6.7%Ni/meso-SiO_2_ are poorly dispersed nickel oxide particles on the silica surface, which is consistent with the XRD and TEM results shown in Figures 1 and 2, respectively.

**Figure 6.**
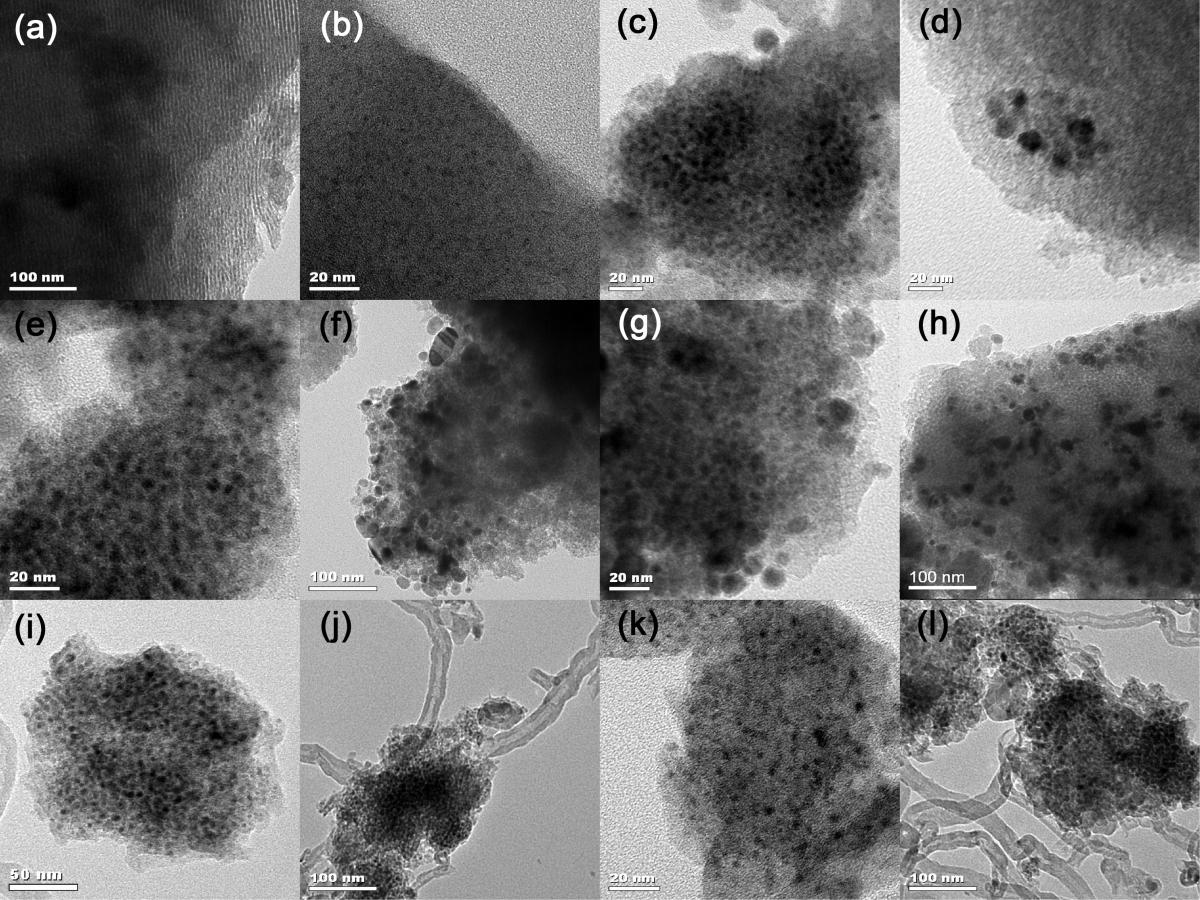
TEM images of (**a**–**h**) the reduced samples and (**i**–**l**) the used samples after catalytic reaction. (**a**) 3.1%Ni-SiO_2_; (**b**) 6.2%Ni-SiO_2_; (**c**,**d**,**i**,**j**) 6.7%Ni-SiO_2_; (**e**,**f**,**k**,**l**) 8.8%Ni-SiO_2_; (**g**) 13.2%Ni-SiO_2_; (**h**) 6.7%Ni/meso-SiO_2_.

From the H_2_ chemisorption results, an increase in metal particle size along with a decrease in dispersion are observed with increasing nickel content in the reduced Ni-SiO_2_ samples. Moreover, a TEM study was carried out on the reduced Ni/meso-SiO_2_ and Ni-SiO_2_ samples to identify the nickel particle size and distribution (Figure 6). No Ni particle was observed for the reduced 3.1%Ni-SiO_2_ sample (Figure 6a), indicating that Ni species are very small and highly dispersed. The nickel particles of the reduced 6.2%Ni-SiO_2_ sample are also well dispersed and estimated to be uniformly 2.5 nm (Figure 6b). Besides the highly dispersed nickel particles with an average particle size of 4.2 nm (Figure 6c), a small amount of isolated nickel particles with a size of about 10–15 nm (Figure 6d) can also be observed on the reduced 6.7%Ni-SiO_2_ sample. When the nickel content is further increased, a large amount of isolated nickel particles with increased particle size are observed on the reduced 8.8%Ni-SiO_2_ sample (Figure 6f). Additionally, some agglomerates outside the silica framework were observed on the reduced 13.2%Ni-SiO_2_ sample (Figure 6g). However, 6.7%Ni/meso-SiO_2_ shows a much larger particle size and worse dispersion in Table 1 and Figure 6h.

### 2.2. Catalytic Performance

**Figure 7.**
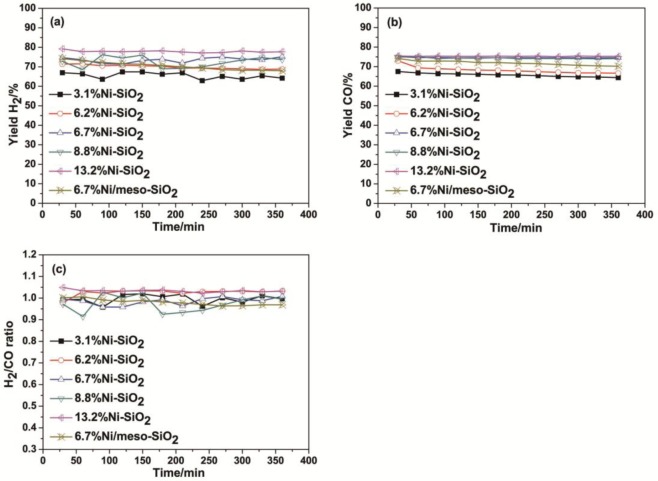
Effect of nickel content on the yields of the (**a**) H_2_, (**b**) CO and (**c**) H_2_/CO ratio in the dry reforming of methane with carbon dioxide at 650 °C and a gas hourly space velocity (GHSV) of 24,000 mL·h^−1^·g^−1^.

Figure 7 shows the initial 6 h catalytic performances of Ni/meso-SiO_2_ and Ni-SiO_2_ catalysts at 650 °C with various nickel content. The yields of H_2_ and CO increase with increasing Ni content. 6.7%Ni-SiO_2_, 8.8%Ni-SiO_2_ and 13.2%Ni-SiO_2_ catalysts exhibit remarkable catalytic activities. The high initial activity of Ni-SiO_2_ catalysts was attributed to the high dispersion of Ni particles and the large BET surface area. In addition, the H_2_/CO molar ratios for Ni/meso-SiO_2_ and Ni-SiO_2_ catalysts are between 0.9 and 1, which is slightly smaller than the stoichiometric ratio of the dry reforming reaction, attributed to the simultaneous presence of the reverse water gas shift reaction [1,6].

**Figure 8.**
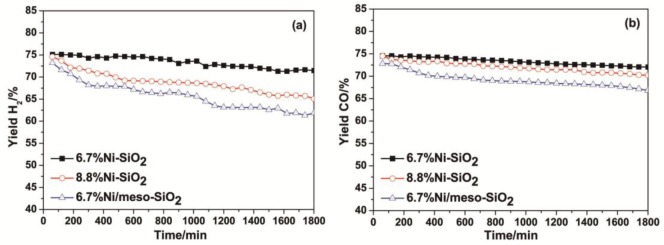
Catalytic stability test for 6.7%Ni-SiO_2_, 8.8%Ni-SiO_2_ and 6.7%Ni/meso-SiO_2_ in the dry reforming of methane with carbon dioxide at 650 °C for 30 h with a GHSV of 24,000 mL·h^−1^·g^−1^.

Considering some nickel agglomerates formed outside the silica framework of 13.2%Ni-SiO_2_, 6.7%Ni-SiO_2_, 8.8%Ni-SiO_2_ and 6.7%Ni/meso-SiO_2_ were chosen to test the stability in 30 h time-on-stream, and the results are shown in Figure 8. It can be seen that there was almost no significant activity loss over 6.7%Ni-SiO_2_. A slight decrease in the yield of H_2_ (*ca*. 10%) and CO (*ca*. 5%) and the deactivation over 8.8%Ni-SiO_2_ were observed, whereas a much faster deactivation can be observed over 6.7%Ni/meso-SiO_2_.

**Figure 9.**
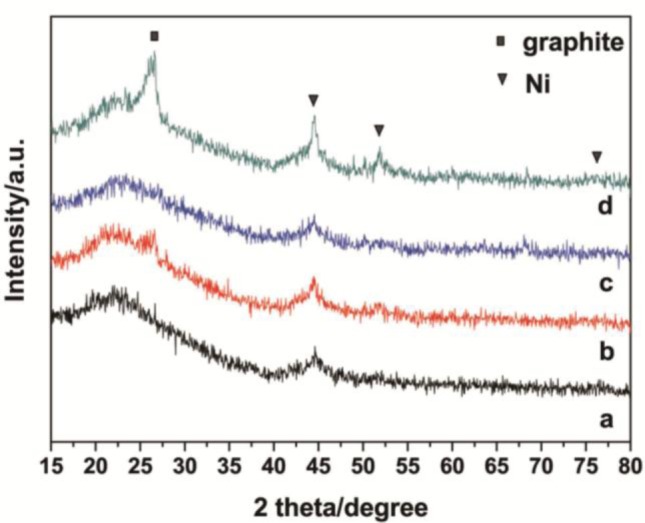
XRD patterns of (**a**,**c**) the reduced and (**b**,**d**)used catalysts. (**a**) and (**b**) 6.7%Ni-SiO_2_; (**c**) and (**d**) 8.8%Ni-SiO_2_.

To obtain more insight into the factors affecting the deactivation of Ni-SiO_2_ catalysts, the used and reduced catalysts were characterized with XRD (Figure 9) and TEM (Figure 6). Distinctly, there are three peaks in the XRD pattern of the used 8.8%Ni-SiO_2_ sample assigned to metallic Ni (JCPD 87-0712), whereas the reduced 8.8%Ni-SiO_2_ sample only has a broad metallic Ni peak around 45°, indicating that a strong sintering occurred over 8.8%Ni-SiO_2_ after reaction. In contrast, the width of the broad metallic Ni peak for the reduced and used 6.7%Ni-SiO_2_ is nearly unchanged, suggesting that the growth of the metallic Ni particles during the dry reforming reaction was almost prevented. Furthermore, the diffraction peak at 26° is assigned to graphitic carbon. This graphite diffraction that appears over the used catalysts is more pronounced for the used 8.8%Ni-SiO_2_ sample. This result can be confirmed by the TEM images (Figure 6).

**Figure 10.**
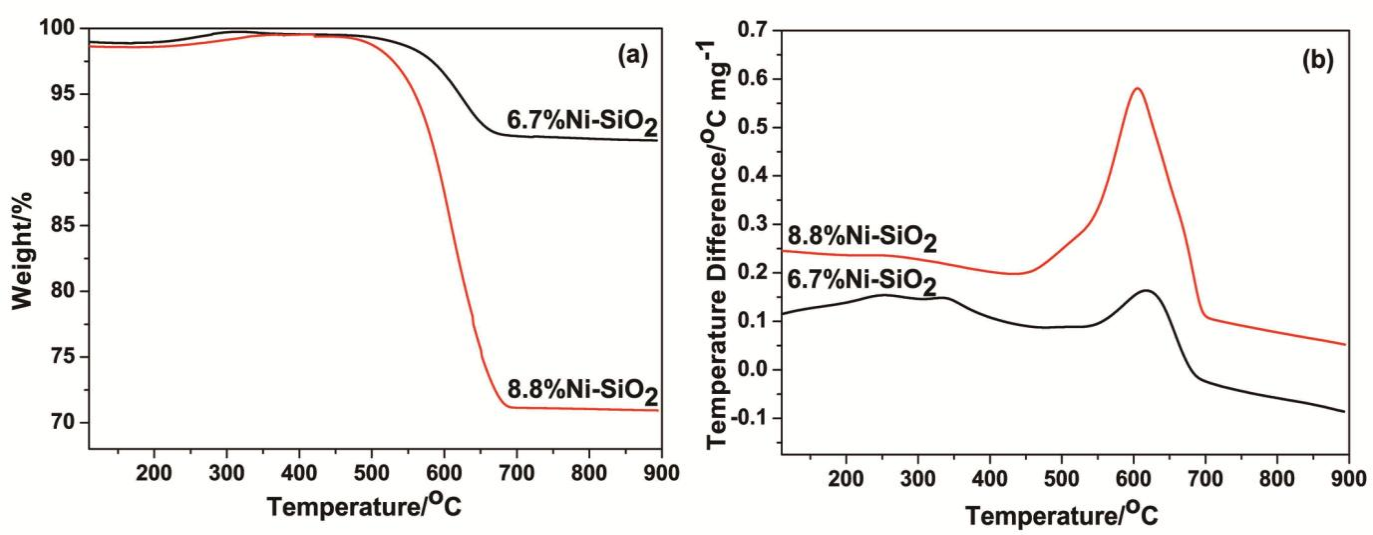
(**a**) The TG and (**b**) DTA profiles of the used catalysts after the 30 h reaction.

The amount of deposited carbon on the used catalysts after reaction for 30 h was also calculated from thermogravimetric (TG) profiles, which are shown in Figure 10a. The TG curve initially experienced a slight rise in the region from 110 °C to 400 °C, which is probably derived from the oxidation of metallic Ni particles. The weight losses from 400 °C to 700 °C are caused by the oxidation of graphitic carbon [30]. It could be clearly observed that the weight loss of the carbon deposition over the used 8.8%Ni-SiO_2_ catalyst (27.6%) was much higher than that over the used 6.7%Ni-SiO_2_ catalyst (7.4%), in agreement with the results of XRD and TEM (Figure 9 and Figure 6).

Due to the oxidation of carbon deposits on the catalyst surface, exothermal peaks can be observed in the DTA profiles. It is well known that a much higher temperature is required to oxidize the graphitic carbons compared to the amorphous carbons [1,23]. In the DTA study, it can be found in Figure 10b that there is a significant exothermic peak around 600 °C over the used 6.7%Ni-SiO_2_ and 8.8%Ni-SiO_2_ catalysts. This peak can be attributed to the oxidation of the graphitic carbons. Additionally, a small exothermic peak observed at relatively low temperature over the used 6.7%Ni-SiO_2_ catalyst suggests that a small amount of the carbon species over the used 6.7%Ni-SiO_2_ catalyst are amorphous carbons.

From the results obtained, it is indicated that the sintering of metal particles and carbon deposition are important factors affecting the catalyst performance in DRM. It was believed that smaller Ni particles possessed an enhanced capacity to inhibit the carbon deposition [9,18,19,32]. The large isolated nickel particles in the used 8.8%Ni-SiO_2_ catalyst are responsible for the formation of a large amount of carbon, which resulted in the poor stability of the catalyst [33,34].

### 2.3. The Anchoring Effect

**Figure 11.**
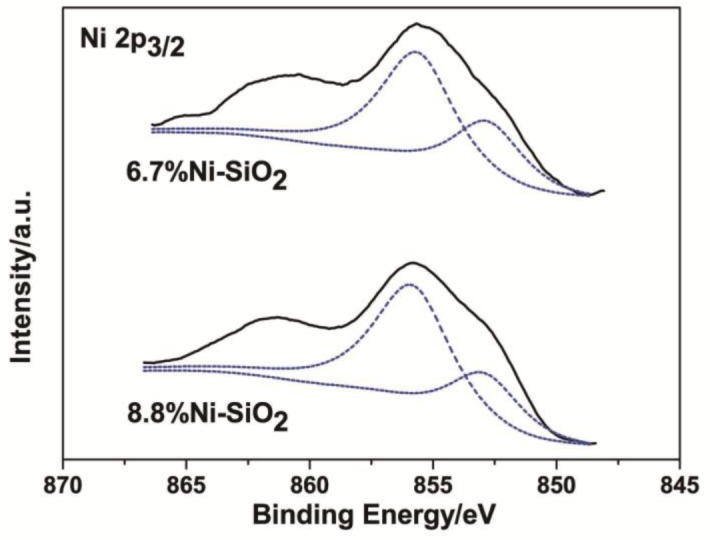
XPS profiles of Ni 2p_3/2_ for the reduced 6.7%Ni-SiO_2_ and 8.8%Ni-SiO_2_ samples.

The nature of surface Ni and Si of the reduced 6.7%Ni-SiO_2_ and 8.8%Ni-SiO_2_ were studied by X-ray photoelectron spectroscopy (XPS) (Figure 11). The 2p_3/2_ peak of Ni was chosen to characterize the chemical state of nickel (Table 2). The reduced 6.7%Ni-SiO_2_ shows the main line of Ni 2p_3/2_ at 855.6 eV, indicating the presence of Ni^2+^ species [18,26]. Furthermore, compared to NiO (854.6 eV), higher binding energy implies that Ni^2+^ does not exist in the form of free NiO, and a strong interaction between Ni^2+^ species and the SiO_2_ framework had been formed [35,36]. This fact is consistent with the FT-IR results, that part of the Si could be substituted by Ni^2+^ in the silica framework. Apart from this peak, the reduced sample showed another shoulder of Ni 2p_3/2_ at 852.8 eV, which can be associated with metallic Ni on the surface [36,37]. The presence of Ni^2+^ and Ni° species indicates that the sample was only partly reduced during the reduction treatment. For the reduced 8.8%Ni-SiO_2_, the binding energies are similar to the reduced 6.7%Ni-SiO_2_. In addition, both spectra show primary satellite peaks around 862.0 eV, which should be due to the shake-up electrons [30]. It can be found in Table 1 that only part of Ni was reduced for all samples. Regarding the extent of the reduction of Ni, it can be observed that the values estimated from Table 1 are close to the ones determined from XPS in Table 2.

The anchoring effect was assumed to be due to the direct contact between metallic clusters and unreduced or partially reduced metal ions within the SiO_2_ matrix on the wall surface [23,38]. Therefore, it is postulated that the anchoring effect between metallic Ni particles and Ni ions distributed in the silica matrix is responsible for the stabilization of the metallic Ni nanoparticles. Bonneviot *et al*. [38] described the anchoring sites in Ni/SiO_2_ catalyst. They found that sintering can be inhibited, because the Ni ions acted as chemical anchors. On the basis of the XPS results, it is likely that Ni^2+^ ions in the silica matrix act as anchoring sites.

As reported [24,25,39], unconfined metallic clusters can freely migrate and aggregate to produce larger particles, but the anchored clusters almost retain their initial size as a result of the constraint of the anchoring sites. TEM results of the reduced and used 6.7%Ni-SiO_2_ shown in Figure 6 indicate that the particle size of metal nickel dispersed on silica matrix remains stable during the reaction, resulting from the existence of the anchoring effect. In addition, the metal sintering occurring during the reaction over 8.8%Ni-SiO_2_ leads to the significant carbon deposition.

Combining the results of the catalytic test and the nickel particle size, it is manifested that small Ni particles are beneficial to the catalytic performance. A similar trend that correlated with metal particle size and catalytic performance was also observed over Ni-GRF, Ni/SiO_2_ and Ni/MgO catalysts [25,40,41]. The excellent anti-deactivation properties and high catalytic efficiency of the 6.7%Ni-SiO_2_ sample can be associated with the anchoring of small metallic Ni particles by nickel ions located within the silica matrix.

## 3. Experimental Section

### 3.1. Catalyst Preparation

Five samples with different nickel contents were prepared by the evaporation-induced self-assembly method. Nickel nitrate (Ni(NO_3_)_2_·6H_2_O), ethanol, tetraethyl orthosilicate (TEOS, Si(OC_2_H_5_)_4_), triblock copolymer P123 (EO_20_PO_70_EO_20_) and ammonia were used as reactants. The molar ratio of TEOS:P123:ethanol:Ni(NO_3_)_2_:NH_3_:H_2_O was 1:0.017:64.4:(0.051–0.205):0.054:12.9. The resulting sol was transferred onto a glass petri dish and aged at 40 °C under a controllable relative humidity of *ca*. 45% for 2 days to obtain a gel product. The as-made sample was calcined at 400 °C for 2 h in air to remove the block copolymer surfactant. The samples prepared were denoted as *x*Ni-SiO_2_, where *x* is the mass content of nickel being 3.1%, 6.2%, 6.7%, 8.8% or 13.2%. For comparison, pure mesoporous silica (meso-SiO_2_) was prepared by the above method without the addition of a nickel source.

Ni/meso-SiO_2_ was prepared by the wetness impregnation method as a reference using Ni(NO_3_)_2_·6H_2_O as the precursor. After impregnation, the sample was dried at 100 °C overnight and calcined in air at 400 °C for 2 h. The sample was denoted as 6.7%Ni/meso-SiO_2_, in which the mass content of Ni was 6.7%.

### 3.2. Characterization

XRD patterns were recorded on a Bruker D8 Advances X-ray diffractometer using Cu-Kα1 radiation (λ = 0.15406 nm) with a voltage of 40 kV and a current of 40 mA. The average crystallite size of metallic Ni in the reduced catalysts was calculated using the Scherrer equation. N_2_ adsorption-desorption isotherms were obtained at −196 °C using a Micromeritics Tristar 3000 apparatus. Element analysis was carried out on a Thermo Elemental IRIS Intrepid inductively coupled plasma atomic emission spectrometer and an Elementar Vario EL III microanalyzer. FT-IR spectra were recorded on a Nicolet Nexus 470 infrared spectrometer using KBr discs. TPR studies were performed on a Micromeritics ChemiSorb 2720 apparatus. TPR profiles were obtained by passing a 10% H_2_/Ar flow (50 mL/min) through the sample. Temperature was increased at a rate of 10 °C/min from room temperature to 1000 °C, and the amount of H_2_ consumed was determined with a thermal conductivity detector (TCD). TEM images were obtained from a JEOL JEM2011 microscope operated at 200 kV. At least 10 representative images were taken for each sample. In order to obtain statistically reliable information, the size of *ca*. 200 particles was measured. XPS was carried out on a Versa Probe PHI 5000 spectrometer utilizing an Al Kα (hυ = 1486.6 eV) X-ray source. Hydrogen chemisorption was performed at 35 °C with a Micromeritics 2750 chemisorption system. All the catalysts were reduced at 650 °C in pure H_2_ flow for 2 h prior to the measurement. The amounts of carbon formed on the catalysts were determined by a Perkin Elmer TGA7 thermogravimetric instrument in air with a flow rate of 50 mL/min and a heating rate of 10 °C/min.

### 3.3. Catalytic Experiments

The dry reforming experiments were conducted at atmospheric pressure in a conventional flow apparatus using a stainless steel fixed bed reactor with quartz lining with inside diameter of 5 mm. Typically, 30 mg of the catalyst with 40–60 mesh was reduced in hydrogen at 650 °C for 2 h before reaction. The CO_2_/CH_4_ reforming was carried out at 650 °C with a gas hourly space velocity (GHSV) of 24,000 mL·h^−1^·g^−1^ (CH_4_:CO_2_:N_2_ = 1:1:3). The effluent was analyzed using on-line gas chromatography with a TCD. A TDX-01 column was used for the separation of H_2_, CO, CH_4_ and CO_2_. The conversion of CH_4_ and CO_2_, the yields of H_2_ and CO, as well as the ratio of H_2_/CO are defined as follows:

$$\begin{array}{c} \text{Conversion}({\mathrm{CH}}_{4})=\frac{{\left(F_{{\mathrm{CH}}_{4}}\right)}_{\text{in}}-{\left(F_{{\mathrm{CH}}_{4}}\right)}_{\text{out}}}{{\left(F_{{\mathrm{CH}}_{4}}\right)}_{\text{in}}}\times 100\%\\ \text{Conversion}({\mathrm{CO}}_{\text{2}})=\frac{{\left(F_{{\mathrm{CO}}_{2}}\right)}_{\text{in}}-{\left(F_{{\mathrm{CO}}_{2}}\right)}_{\text{out}}}{{\left(F_{{\mathrm{CO}}_{2}}\right)}_{\text{in}}}\times 100\%\\ \text{Yield}(H_{\text{2}})=\frac{{\left(F_{H_{\text{2}}}\right)}_{\text{out}}}{2\times {\left(F_{{\mathrm{CH}}_{\text{4}}}\right)}_{\text{in}}}\times 100\%\\ \text{Yield}(\mathrm{CO})=\frac{{\left(F_{\mathrm{CO}}\right)}_{\text{out}}}{{\left(F_{{\mathrm{CH}}_{\text{4}}}\right)}_{\text{in}}+{\left(F_{{\mathrm{CO}}_{\text{2}}}\right)}_{\text{in}}}\times 100\%\\ \frac{H_{\text{2}}}{\mathrm{CO}}=\frac{{\left(F_{H_{\text{2}}}\right)}_{\text{out}}}{{\left(F_{\mathrm{CO}}\right)}_{\text{out}}} \end{array}$$

where ${\left(F_{{\mathrm{CH}}_{\text{4}}}\right)}_{\text{in}}$ and ${\left(F_{{\mathrm{CO}}_{\text{2}}}\right)}_{\text{in}}$ are inlet flow rates and ${\left(F_{{\mathrm{CH}}_{\text{4}}}\right)}_{\text{out}}$, ${\left(F_{{\mathrm{CO}}_{\text{2}}}\right)}_{\text{out}}$, ${\left(F_{H_{\text{2}}}\right)}_{\text{out}}$ and ${\left(F_{\mathrm{CO}}\right)}_{\text{out}}$ are outlet flow rates.

## 4. Conclusions

A series of Ni-SiO_2_ samples with different nickel content (3.1%–13.2%) was synthesized by the evaporation-induced self-assembly method, which leads to the homogeneous incorporation of nickel species into the mesoporous silica matrix under low Ni content. After H_2_ reduction, the catalytic activity of these samples was tested in the carbon dioxide reforming of methane. Nickel content and nickel particle size have significant effects on the yields of H_2_ and CO. It was found that the 6.7%Ni-SiO_2_ sample had the highest catalytic activity and stability, which is attributed to the formation of small and well-dispersed Ni particles, due to the anchoring effect between metallic Ni particles and unreduced Ni ions within the silica matrix.

## Figures and Tables

**Table 1.** Physiochemical properties of the meso-SiO_2_, Ni/meso-SiO_2_ and Ni-SiO_2_ samples. TPR, temperature-programmed reduction.

Sample	Surface area (m^2^·g^−1^)	Pore volume (cm^3^·g^−1^)	Pore diameter (nm)	TPR results ^a^ (%)	Ni ^b^ (wt%)	Reduced Ni ^c^ (wt%)	Ni dispersion ^d^ (%)	Metal particle size ^e^ (nm)
meso-SiO_2_	584	0.71	4.13	–	–	–	–	–
6.7%Ni/meso-SiO_2_	460	0.54	4.10	–	6.7	4.4	4.7	21.4
3.1%Ni-SiO_2_	504	0.62	3.60	–	3.1	0.4	24.8	4.1
6.2%Ni-SiO_2_	484	0.57	3.58	–	6.2	3.0	16.3	6.2
6.7%Ni-SiO_2_	479	0.46	3.57	7.1	6.7	3.0	13.1	7.7
8.8%Ni-SiO_2_	419	0.38	3.58	9.8	8.8	3.3	9.6	10.5
13.2%Ni-SiO_2_	466	0.39	3.55	23.8	13.2	6.5	7.2	14.0

a H_2_ consumed in the range of 250–350 °C/total consumed H_2_;

b measured by inductively coupled plasma atomic emission spectroscopy;

c the weight percentage of reduced Ni in the reduced samples based on the increased mass determined by thermogravimetry (TG);

d calculated assuming H_ad_/Ni_surf_ = 1;

e the metal particle shape is assumed to be spherical, and the metal particle size of the reduced samples is determined by H_2_ chemisorption.

**Table 2.** Binding energies of Ni 2p_3/2_ and the percentage of Ni^2+^ and Ni° for the reduced 6.7%Ni-SiO_2_ and 8.8%Ni-SiO_2_ samples.

Sample	Ni^2+^		Ni°	
	Ni 2P_3/2_ (eV)	Ni^2+^/(Ni^2+^ + Ni°)	Ni 2P_3/2_ (eV)	Ni°/(Ni^2+^ + Ni°)
6.7%Ni-SiO_2_	855.6	62%	852.8	38%
8.8%Ni-SiO_2_	855.8	66%	852.9	34%
